# Multi-functional imaging inspired by insect stereopsis

**DOI:** 10.1038/s44172-022-00039-y

**Published:** 2022-11-30

**Authors:** Kisoo Kim, Kyung-Won Jang, Sang-In Bae, Ki-Hun Jeong

**Affiliations:** 1 Department of Bio and Brain Engineering, Korea Advanced Institute of Science and Technology (KAIST), 291 Daehak-ro, Yuseong-gu, Daejeon, 34141 Republic of Korea; 2 KAIST Institute for Health Science and Technology, KAIST, Daejeon, 34141 Republic of Korea; 3 Intelligent Optical Module Research Center, Korea Photonics Technology Institute (KOPTI), 9, Cheomdan Venture-ro 108beon-gil, Buk-gu, Gwangju, 61007 Republic of Korea

**Keywords:** Imaging and sensing, Micro-optics

## Abstract

Insects exhibit intriguing vision capability using fragmented information from arrays of lenses. Visual disparity between lenses or eyelets (also known as stereopsis) helps insects to locate their prey or find landing spots. Here we report a multi-functional imaging system inspired by insect stereopsis using a single ultrathin microlens array camera. Individual channels through tens of microlenses capture distinct images with visual disparities. We demonstrate that reconstruction of these images can provide diverse capabilities, such as simultaneous near-distance microscopic imaging, high speed imaging at far distances and 3D depth imaging at intermediate distances. Our findings offer clues to further understand the vision capability of insects as well as insights for advanced compact functional imaging tools.

## Abstract (web summary)

An imaging device inspired by insect stereopsis allows for simultaneous near-distance microscopic imaging, high speed imaging at far distance and 3D depth imaging at intermediate distances. The camera, reported by Kisoo Kim and colleagues, gives clues as to how insects see the world and offers insights for designing compact cameras with multifunctional capabilities.

## Introduction

Stereopsis provides a crucial feature for visual perception in detecting spatial and temporal variations^1–4^. Insect stereopsis performs diverse functions such as prey hunting^5,6^, navigation^3,7^, and predator evasion^8^ with tiny visual organs of eyelets. For instance, praying mantises exert stereoscopic depth perception for striking at a target^9,10^, or dragonflies utilize optic flow stimuli for mitigating collision^9,11,12^. This visual perception often depends on their eyelet structures and visual processing for the channel images of individual eyelets^13,14^. Visual stereopsis of apposition or superposition compound eyes mostly found in nature offers relatively inaccurate depth information because a small number of photoreceptor cells in a single facet lens generate a simple point image^13–16^. However, the unusual design of Xenos peckii eyes exhibits multi-view stereopsis with high visual acuity through chunk sampled images, which is created by multiple photoreceptors on a single facet lens^17–19^.

Like insect’s vision, multi-aperture imaging systems capture an array of scenes with the visual parallax of a target object in a single shot^20–24^. They allow stereoscopic imaging by reconstructing spatiotemporal data^25–28^. However, some technical limitations still exist in emulating insects’ visual features due to their limited depth-of-field and bulky systems^29,30^. A compact camera with a dimension similar to that of an insect eye is required to precisely investigate the characteristics of an insect’s vision. Compound eye cameras mimicking the visual structures of insects also collect visual information with the visual parallax at an extremely small scale. They allow wide field-of-view (FOV) and optic flow imaging through the visual disparities of microlens arrays^12,31–34^. The main technical bottleneck still remains in the low image resolution, which limits the exploration of various functional imaging of insect vision^16,35^. Recently, an ultrathin array camera provided high-resolution imaging by emulating the visual structures of *Xenos peckii* on a flat image sensor^19^. All the previous works have only demonstrated compound eye cameras structurally similar to insects; however, the inquiry of diverse insect vision still remains and requires further exploration to understand an insect eye’s visual principles in overall viewing planes. Multifunctional imaging at various distances is required to understand insect stereopsis instead of fixed target imaging.

**Fig. 1:**
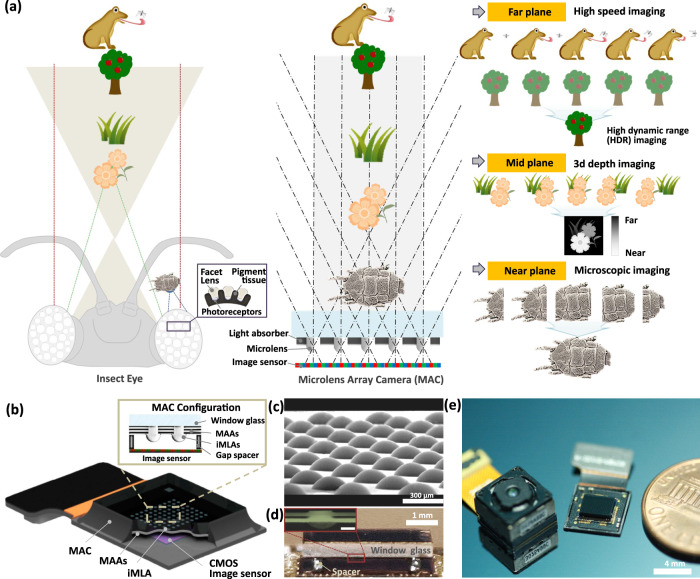
Biological inspiration from the stereopsis of insect. **a** Comparison of stereopsis between insect eyes and a versatile ultrathin microlens array camera (MAC) depending on a target object distance. Like visual disparities of insect eyes, the ultrathin array camera captures assorted functional images such as a microscopic image in the near object plane, a 3D depth map in the mid plane, and a high dynamic range (HDR) image as well as high-speed images in the far object plane. **b** A schematic illustration of MAC configuration. Integrated configuration of MAC consisting of a lens plate with inverted microlens arrays (iMLA), multilayer aperture arrays (MAAs), and alumina gap spacers on an active pixel image sensor. **c** Scanning electron microscopic (SEM) image of microlens arrays. **d** Side and **e** perspective view photographs of fully-packaged ultrathin camera. The inset indicates the cross-sectional microscopic image of microlens with the MAAs, and the scale bar is 100 μm.

Here we report multi-functional imaging inspired by insect stereopsis using a single ultrathin microlens array camera (MAC). The comparative stereopsis of insect eyes and MAC is explained by their optical structures and three different viewing planes depending on a target distance (Fig. 1a, Table 1). Insects often perceive an object distance from the parallax cues of visual field overlaps obtained from each eyelet and show infinite depth-of-field^6,13,14^. Likewise, the MAC has a constant FOV for microlens arrays and captures partial images with all-in-focus, so the visual parallax between channel images varies with the object distance. For instance, the individual channels of MAC capture the close-up partial images for each channel on the near plane, which can be stitched for wide-field microscopic imaging. Close-up imaging without image blur is possible because of the short focal length and the ultracompact dimension of the MAC. They also recognize the array images of an object with clear visual disparities on the mid plane, reconstructing 3D depth maps. In addition, they also obtain similar scenes without visual disparities between individual channels on the far plane, which can offer a single high-dynamic-range (HDR) image after the image reconstruction. In particular, the MAC even offers high-speed imaging on the far plane by using the rapid and sequential image acquisition through the frame fragmentation of a single rolling shutter readout. These multiple functions can be achieved with a single camera of MAC by emulating the visual perception of insects. The distance range of three different planes is controlled by changing the optical parameters such as the channel period and the focal length of the microlens (Supplementary Note 1 and Supplementary Fig. 1). The overlap width and visual disparities of array images are also changed according to the object distances, and an object position can infer through the overlap degree of array images. For example, the close-up partial images for each channel can be observed because the overlap width decreases when the object is positioned close to the lens. The MAC configuration includes a window glass, multilayer-aperture arrays (MAAs), inverted micro-lens arrays (iMLAs), and alumina spacers on a single complementary metal–oxide–semiconductor (CMOS) image sensor (Fig. 1b). The iMLAs with the MAAs allow for efficiently adjusting the viewing angle as well as ultrathin packaging with a short focal length. The measured surface profile indicates the physical dimension of a single microlens (Supplementary Fig. 2). A scanning electron micrograph shows the microfabricated iMLAs (Fig. 1c). The cross-sectional image of MAC demonstrates that the iMLAs with MAAs are fully packaged on a single and flat CMOS image sensor (Fig. 1d). In particular, the captured image of MAC shows an exceptionally ultrathin total track length, compared to a commercial compact single-lens camera (Fig. 1e). The fully-packaged MAC is assembled with an image processing board (Raspberry pi 3 B + , Raspberry Pi Foundation) and fixed to an optical mount for imaging experiments (Supplementary Fig. 3).

**Table 1:** Comparison of performances between insect eye and MAC according to image planes.

	Insect eye	MAC
Near plane	Close-up imaging for prey hunting	Microscopic imaging
Mid plane	Distance measurement for landing	3D depth imaging
	Image integration	High-dynamic-range (HDR) imaging
Far plane	High motion sensitivity for predator evasion	High-speed imaging

## Results and discussion

**Fig. 2:**
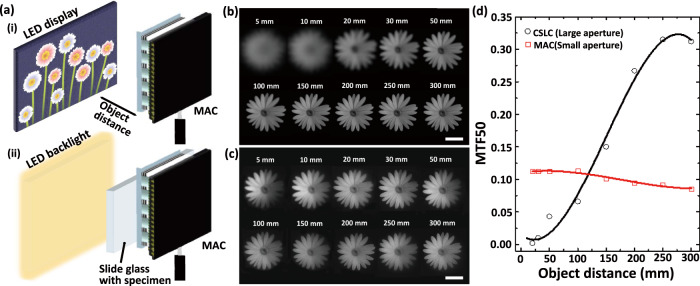
All-in-focus imaging of microlens array camera (MAC). **a** Schematics representing experimental setups for (I) imaging according to object distances and (II) microscopic imaging. “Flower” images captured by **b** a commercialized single-lens camera (CSLC) and **c** the MAC according to the object distance. The “flower” image was displayed on an LED panel. The scale bars are 2 cm. **d** Comparison of modulation transfer function (MTF) 50 between the images from the large aperture and small aperture camera, which demonstrates that images acquired from the small aperture camera have high MTF values within 100 mm object distance than the counterpart.

**Fig. 3:**
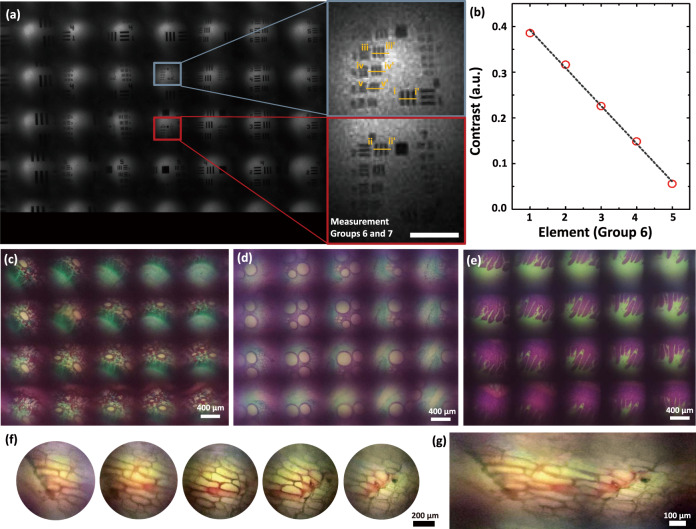
Microscopic imaging of microlens array camera (MAC) in the near object plane. **a** The captured array images of the 1951 USAF test chart. The bars in group 6- element 2 are clearly visible. The scale bar is 10 μm. **b** The measured contrast according to element numbers in group 6. For example, the corresponding resolution of group 6-element 2 with contrast over 0.3 is 6.96 μm. Captured microscopic array images of **c** pine tree stem, **d** pumpkin stem, **e** mouse small intestine, and **f** red onion epidermal cells through the MAC in the near plane. **g** A stitched panoramic image from the images of individual channels.

The optical and imaging qualities of MAC were quantitatively evaluated using the optical sectioning and the modulation transfer function (MTF) measurement of channel images. The MAC provides clear images by precisely placing a CMOS image sensor at the focal length of iMLAs within the depth-of-focus (DOF) using alumina spacers with epoxy glue. The light propagation through iMLAs was optically sectioned using a confocal laser scanning microscope (CLSM, LSM 510, Zeiss) coupled with a laser beam of 532 nm in wavelength (Supplementary Fig. 4). The light from the external laser source is vertically transmitted through the microlens, and the optical beam section is visualized by stacking z-section slices to observe a spot diameter and DOF. The measured focal diameter and the DOF of individual microlenses are 1.32 μm and 19.8 μm in full width at half maximum (FWHM), respectively. Note that the thickness error of alumina spacers with epoxy glue is 12 μm on average, which satisfies the package tolerance less than the DOF of iMLAs (Supplementary Fig. 5). A target object of the Siemens star was captured from the MACs of different microlens diameters under the same f-number (F/1.7, see Supplementary Fig. 6). The experimental results clearly show that the relative illumination, i.e., changes in the illumination from the center to the outside, and the inverse MTF (iMTF) logarithmically increase with the microlens diameter. However, both values become saturated as the microlens diameter increases, and the total track length of MAC increases linearly. As a result, the microlens diameter of 150 μm was finally selected for the versatile ultrathin camera (iMTF50 = 9.63 μm, total track length = 810 μm). The microlens diameter was also considered through the trade-off relationship of multifunctional imaging by characterization at multi-view planes according to various diameters. The short focal length of the microlens not only allows all-in-focus imaging from the near to the far planes but also substantially reduces the minimum object distance (MOD), i.e., the nearest target location from a camera to acquire a clear image (Fig. 2a and Supplementary Fig. 7). A conventional single-lens camera (CSLC, raspberry pi camera module V2, F/2, DLENS: 1.5 mm) with a large aperture captures blurred images at close object distances (Fig. 2b). However, the MAC provides clear images for all object distances (Fig. 2c). The measured MTF50 of the MAC is distinctly higher than that of CSLC when the object distance is less than 130 mm (Fig. 2d). The MAC clearly resolves the bars in group 6-element 2 of the 1951 U.S. Air Force (USAF) resolution chart, corresponding to the image resolution of 6.96 μm (Fig. 3a, b).

The window glass thickness of the demonstrated MAC is 500 μm, and the magnification can be further enlarged by optimizing the glass thickness and by adjusting the lens and observation distance. Each channel of MAC observes the partial images of a target object with the same FOV at the near target distance, and the partial images can be integrated into a single image through image stitching. The image resolution also allows microscopic observation of microstructures such as the pine tree stem and red onion epidermal cells at the distance of total track length, where the sample is in contact with the top glass window of MAC (Fig. 3c–f). The multiple channel images are then stitched to form a single panoramic image (Fig. 3g). The sequence of channel images is revered for the acquisition of the stitched image because each lens captures reverse images.

**Fig. 4:**
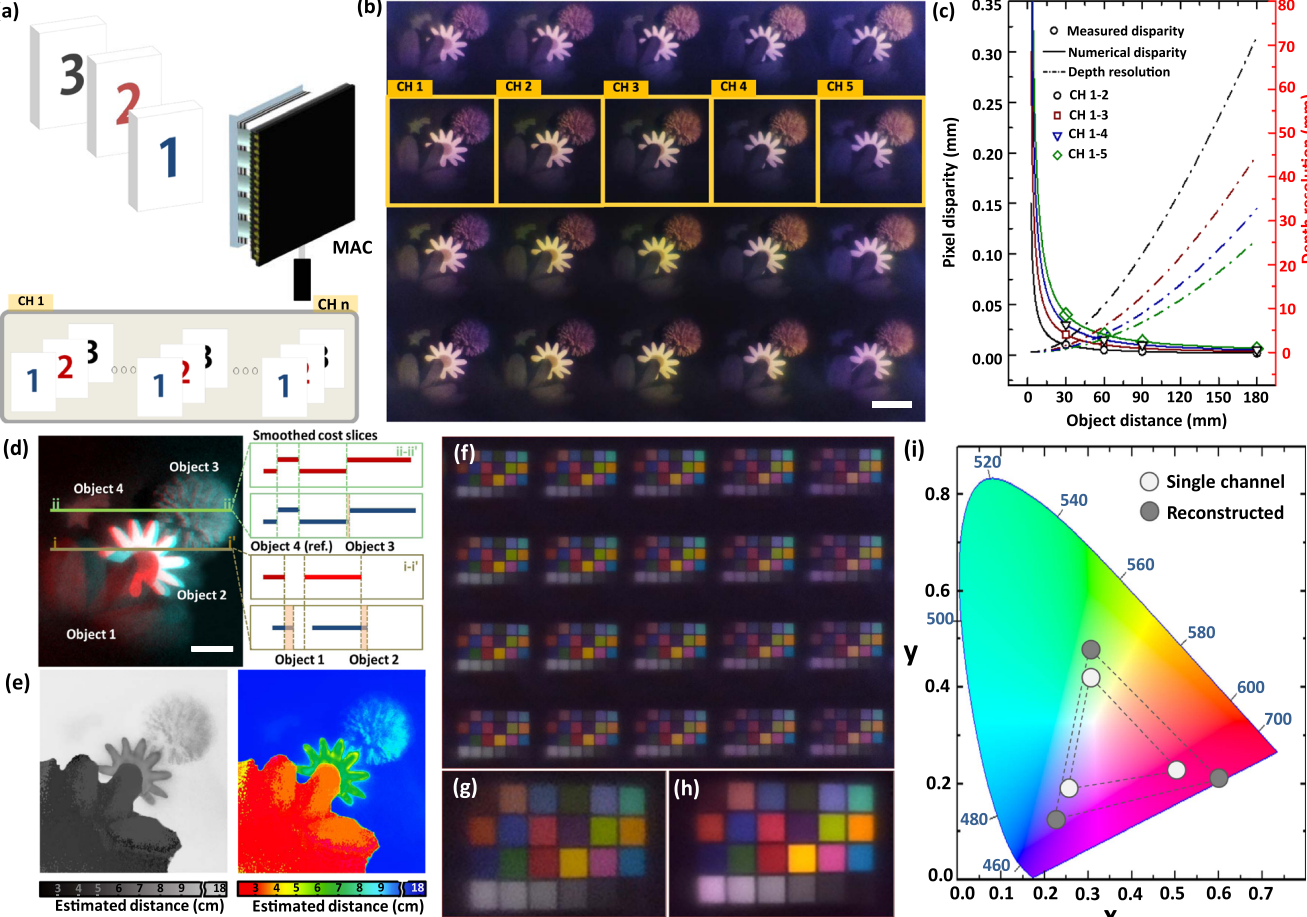
3D depth and high dynamic range (HDR) imaging by using the microlens array camera (MAC). **a** A schematic diagram of 3D depth imaging by using a visual disparity between each channel image. **b** The captured array images of flowers and a star at different depths. The overlap differences between floral disc and the first flower leaf clearly demonstrate the different perspectives of each channel. The scale bar represents the size of an object located at a 6 cm distance and is 4 cm. **c** Pixel disparity and depth resolution depending on the object distance and the channel periods. The scale bar is 2 cm at a 6 cm object distance. **d** Overlapped image of individual channel images by using a red-cyan anaglyph and corresponding smoothed cost slices. The disparity was calculated by using the difference between the slice edges of red and cyan images corresponding to each object. **e** Reconstructed 3D depth map images corresponding to the red-cyan anaglyph image (Left: grayscale map, Right: pseudo color map). **f** HDR imaging from the array images of color checker chart. **g** An image from the single channel of MAC. **h** A reconstructed image by HDR merge. **i** The CIE-1931 chromaticity diagram representing the measured spectra of single channel image and reconstructed image.

The MAC extracts the depth information of objects through the stereopsis in the mid-plane. The visual parallax of individual channel images provides 3D depth information as well (Fig. 4a). The experimental results clearly demonstrate that the 3D model of flowers and a star was captured as channel images with different viewing directions (Fig. 4b). The pixel disparities, i.e., the distances between two corresponding points between individual channel images, and their corresponding depth resolution were also calculated according to the channel period and the object distance through image overlapping (Fig. 4c). The experimental results show that the pixel disparities exponentially increase as either the object distance reduces or the channel period increases. The depth resolution was determined by the object depth when the difference in the pixel disparity between two points corresponds to the image sensor pixel size. As a result, the depth resolution increases by reducing the object distance or increasing the channel period. The calculated depth resolutions of a 3D object are 3.7 μm and 790 μm for 2.5 mm and 30 mm in the distance, respectively. The red-cyan anaglyph image was further visualized by overlapping the individual channel images captured at both ends of iMLAs according to the target distance of 3D models (Fig. 4d). The corresponding 3D depth maps were finally reconstructed using the depth map generation algorithm with fast cost-volume filtering (Fig. 4e)^36^. In particular, the depth map and the pixel disparities are shown in both *x* and *y* axes due to the 2D arrangement of iMLAs (Supplementary Fig. 8). The individual channels of MAC observe the same image of a target object in the far object plane due to the same viewing direction (Fig. 4f). As a result, the reconstruction of array images captured from the far plane delivers a single image with exceptionally high contrast using the HDR merge algorithm (Fig. 4g, h). The reconstructed image shows not only the HDR for grayscale level over 1.61 times but also a color space of gamut expanded by a factor of 2.13 times, compared to the corresponding partial image (Fig. 4i and Supplementary Fig. 9). After the HDR image reconstruction, the calculated image contrast is 0.72, which is 1.09 times higher than that of a single-channel image. This value also corresponds to 86% of the image contrast of a CSLC with a comparable lens parameter.

**Fig. 5:**
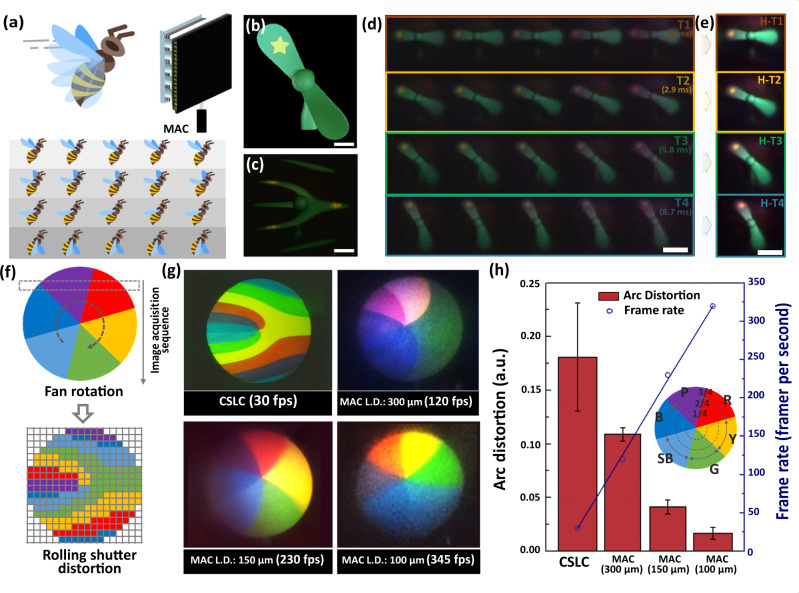
High-speed imaging through the microlens array camera (MAC). **a** Schematic illustrations of capturing a fast-moving object through the MAC. **b** The original photograph of a fan with two wings. The scale bar is 2 cm. **c** The imaging of “a rotating fan” captured by the conventional single-lens camera (CSLC). The image captured through the CSLC shows motion artifacts due to the rolling shutter effect. Scale bar, 3 cm. **d** The array images of “a rotating fan” captured by the MAC. The camera captures different moments in each row of channels without motion artifacts. The scale bar is 8 cm. **e** Reconstructed images of array images in the horizontal direction by using the high dynamic range (HDR) merge. Scale bar, 8 cm. **f** Schematics for image distortion due to the rolling shutter effect. **g** The captured images of a rotating 6-color disc through the CSLC and the MAC. The image distortion is reduced as the lens diameter (L.D.) decreases. Note that colors appear slightly different due to chief ray angle (CRA) mismatching according to lens diameters. **h** The measured arc distortion of each color in the rotating 6-color disc. The error bars represent standard deviations (*n* = 5).

The MAC further allows high-speed imaging by using the frame fragmentation of a single rolling-shutter readout (Supplementary Fig. 10). The CSLC often exhibits motion artifacts when capturing a fast-moving object due to the rolling shutter effect of CMOS image sensors. The image distortion is caused by recording partial images of a whole scene at different instants. However, the array images of MAC capture high-speed scenes without motion artifacts as well as different moments along the vertical direction of iMLAs (Fig. 5a). The MAC forms array images on a single image sensor; thus, the image acquisition time in each channel is shorter than that of CSLC. In this experiment, high-speed images were captured using a rotating fan with a star mark (Fig. 5b). The experimental results clearly demonstrate that the CSLC captures the fan image with severe distortion (Fig. 5c). In contrast, the MAC captures the clear array image of a rotating fan without noticeable distortion in the MAC (Fig. 5d). The individual channels along the column of iMLA capture a partial image every 2.89 ms, during which the fan rotates by 25°. The fan speed calculated from the array images turns out to be 1441 rpm. In addition, the reconstruction of array images in a single row further enhances not only the image contrast but also the image resolution by using the HDR merge algorithm (Fig. 5e). The rolling shutter distortion often occurs when a moving object’s capture speed is faster than an image sensor’s frame rate due to the vertical scanning of the entire scene (Fig. 5f). The motion artifacts of CSLC and MAC were quantitatively compared by imaging a rotating 6-color disc. The experimental results clearly show that the captured image through the MAC is much less distorted than the CSLC (Fig. 5g). The calculated frame rate of MAC corresponds to 345 fps for the microlens diameter of 100 μm. Besides, the measured image distortion, i.e., the difference in the arc length of each color, is significantly reduced and the frame rate increases linearly as the microlens diameter decreases (Fig. 5h). As a result, the MAC achieved 11.5 times faster imaging than the CSLC with a 30 fps CMOS image sensor and more than 90% reduction in image distortion due to the frame fragmentation. The overall experimental results in the multi-view plane imply that the imaging performances of MAC have a trade-off relationship between lens diameters and imaging planes. The MAC is optimized to implement the overall functions in the multi-view plane evenly, but re-optimization can improve the function in a specific plane.

## Conclusions

Inspired by the principle of insect stereopsis, we have successfully demonstrated assorted functional imaging through our ultrathin array camera. The unique configuration of channel arrays offers not only clear all-in-focus imaging but also variable visual disparities of individual channels. The MAC exhibits versatile functional features such as transmission microscopic imaging, 3D depth reconstruction, HDR, or high-speed imaging, depending on the target distance. The experimental results exhibit that reconstructed imaging of array images offers various functions with low-cost cameras and clues to understand the superior insect vision features. This multi-functional imaging, inspired by insect stereopsis, provides insight for advanced compact functional camera applications in healthcare, mobile, and surveillance devices.

## Methods

### Camera fabrication

The MAAs were micro-fabricated by using repetitive photolithography of negative-tone black resin (GMC 1040, Gersteltec) and negative-tone transparent resin (SU-8, MicroChem Corp.) on a 4-in. borosilicate wafer (Supplementary Fig. 11). The plasma surface treatment was performed through an atmospheric plasma system (IHP-1000, Applasma Inc.) to enhance the adhesion between the black photoresist (PR) and transparent PR. Positive-tone resin (AZ9260, MicroChem) was photolithographically defined as cylinder array patterns on the MAAs pattern. An upside-down reflow process melts the cylinder patterns to form the spherical shape of lens on a 180° hot plate. The upside reflow method was performed to fabricate the F/1.7 microlens with high curvature because the upside reflow can improve curvature through gravity. The fabricated lens plate was diced by using a dicing saw. For the camera assembly, a CMOS image sensor (Sony IMX 219) and the microfabricated microlens arrays were packaged by using a flip-chip die bonder. The epoxy adhesive was precisely applied on the image sensor through a micro-dispenser, and alumina spacers were placed on the epoxy adhesive. Several spacers were stacked to match the focal length and the overall height of spacers. The alumina spacers were fabricated in precise sizes through grinding and micro-sawing processes. The epoxy adhesive was further applied on the upside of spacers, and the lens plate with the microlens arrays was placed through a flip-chip bonder. In this process, the lens plate and the active pixel area of image sensor are aligned through the microscope. The ultrathin camera was finally assembled by curing the epoxy adhesive on a 120° hot plate for 30 min.

### Image reconstruction algorithm

The left and right images from both sides of the channels were cropped for the 3D depth reconstruction. A stereo matching algorithm using fast cost-volume filtering was used to acquire a 3D depth map^36^. The cost volume was constructed to express disparity space in images, and the cost volume was smoothed by using bilateral and guided filters. The reference pixels are then allocated to the minimum disparity value, and the disparities from the rectified cost were calculated. A depth map was finally achieved by filling pixels using a weighted median filter. The depth map expressed as a grayscale value was changed to the color gradient map. The HDR image reconstruction was performed by using a freeware graphic editor, Chasys Draw IES. The individual channel images were extracted to create the HDR image at far plane imaging. The extracted channel images were registered by aligning layer offset, and converted into weight maps according to the brightness. The weights in under- and overexposed areas were reduced by weighted blending with Laplacian and Gaussian filters^37^. The individual values of each pixel in the image were combined with the blended weight map through multiplication. All algorithms ran on a desktop computer with Intel Core i5-6600K 3.30 GHz and NVIDIA GeForce GTX 960 after capturing images through the Raspberry pi board. The processing time of 3D depth reconstruction takes 10 s and HDR merge takes 1 s.

### Supplementary information

Supplementary Information

## Data Availability

The datasets generated during and/or analyzed during the current study are available from the corresponding author upon reasonable request.

## Code availability

The codes generated during and/or analyzed during the current study are available from the corresponding author upon reasonable request.
