# Supplementary material for: Multi-functional imaging inspired by insect stereopsis
Source: Commun Eng. 2022 Nov 30;1:39. doi: 10.1038/s44172-022-00039-y (PMC10956018; doi:10.1038/s44172-022-00039-y)
Supplement: Supplementary file 1 — Supplementary Information [file 44172_2022_39_MOESM1_ESM.pdf]

# Supplementary Information

# Multi-functional imaging inspired by insect stereopsis

Kisoo Kim[1,2,3], Kyung-Won Jang[1,2], Sang-In Bae[1,2], and Ki-Hun Jeong[1,2]*

[1]*Department of Bio and Brain Engineering, Korea Advanced Institute of Science and Technology (KAIST), 291 Daehak-ro, Yuseong-gu, Daejeon 34141, Republic of Korea*

[2]*KAIST Institute for Health Science and Technology, KAIST, Daejeon 34141, Republic of Korea*

[3]*Intelligent Optical Module Research Center, Korea Photonics Technology Institute (KOPTI), 9, Cheomdan Venture-ro 108beon-gil, Buk-gu, Gwangju, 61007 Republic of Korea*

**Correspondence to: kjeong@kaist.ac.kr*

## Supplementary Note 1: Principle of ultrathin microlens array camera

Working principle of versatile ultrathin array camera. Supplementary Fig. S1 shows schematics for the working principle of ultrathin microlens array camera (MAC). The difference in viewing direction of each lens leads to visual disparities between images depending on the object distance. This visual disparity can be expressed as follows:

$$\text{disparity} = x - x' = \frac{B \cdot f}{z}$$

where, x and x' are the distance to imaging points corresponding to an object P. B is the distance between microlenses and f is the focal length of microlens. z is the distance from the image plane to the object. When microlenses are arranged in a constant period (d), the distances of the near, mid and far planes introduced in Fig. 1a can be defined as follows:

$$z_{near} = MOD \sim \frac{B \cdot f}{d}$$

$$z_{mid} = \frac{B \cdot f}{d} \sim \frac{d \cdot f}{iMLA\ resolution}$$

$$z_{far} = \frac{d \cdot f}{iMLA\ resolution} \sim Infinity$$

,where d (=2f·tan($FOV_{MLA}$/2)) is the channel width, B is the distance between iMLAs at both ends, and iMLA resolution is the minimum resolution to distinguish the visual disparity, depending on the pixel size of CMOS image sensor. The range of near plane is determined from the MOD to the distance that each lens at both ends captures the same target point. The mid plane range is from the endpoint of near plane to the distance where the visual disparity of each lens becomes smaller than the image sensor pixel size. The extent of far object plane defines from the last position of mid plane to infinity. For instance, each plane distance of 150 μm microlens (F/1.7) shows the near plane of from 1.5 mm to 2.36 mm, the mid plane of from 2.36 mm to 81.96 mm, the far plane of from 81.96 mm to infinity. The calculated channel width

(F/1.7, FOV: 70°) is 357 μm, and the number of pixels in a single channel is 318 × 318. The overlap width between lenses in the observation plane varies on the distance of an object, and the overlap width can be calculated from:

$$Overlap\ \ width = d \cdot (z/f - 1)$$

The total field width for lens arrays X can be expressed by the following equation:

$$X = z \cdot N \cdot \tan(\frac{FOV_{MLA}}{2})$$

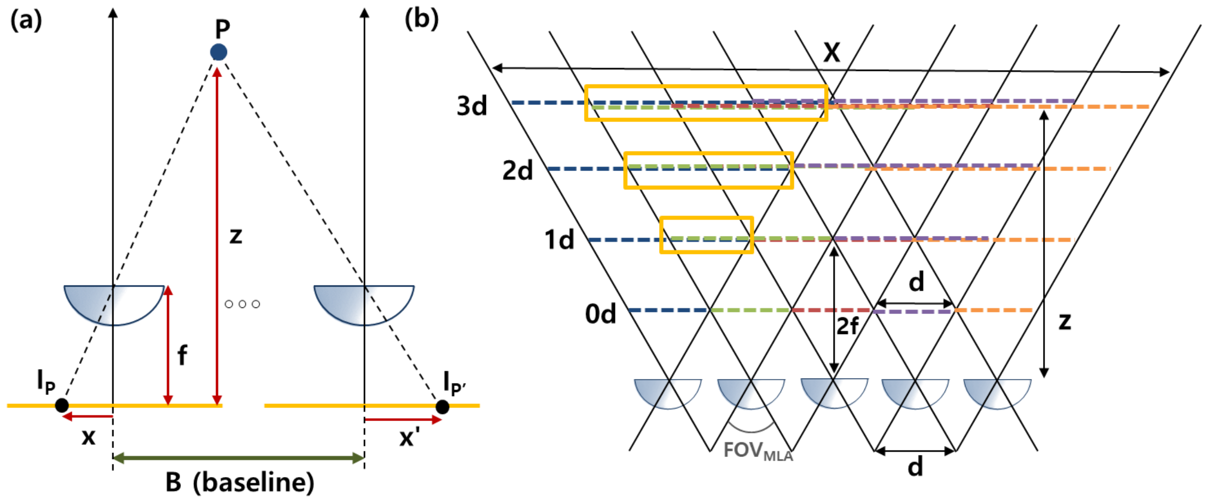


**Supplementary Fig. 1** Parametric design of MAC. (a) Design concept for determining the distances of near, mid and far planes. The distances of each plane are determined by focal length, lens periods and MLA resolution. (b) A design schematic for calculating imaging overlap width. The parameters of lens focal length, object distance, and lens period change the imaging overlap width. The yellow boxes indicate the imaging overlap width between channels. x and x' are the distance to imaging points corresponding to an object P. B is the distance between microlenses and f is the focal length of microlens. z is the distance from the image plane to the object. X is the total field width and d is the channel width.

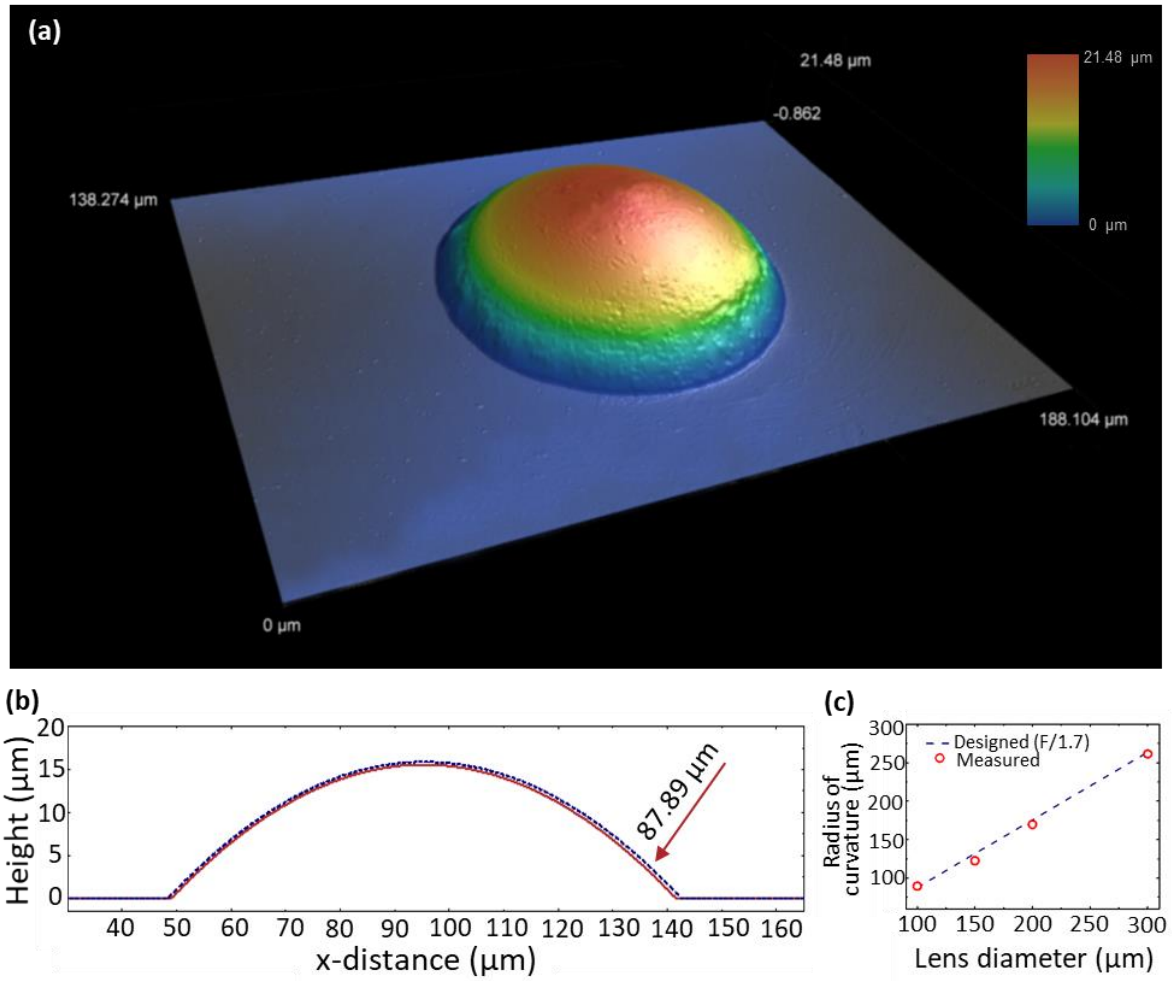


**Supplementary Fig. 2** Radius of curvature of iMLA. (a) Microlens surface shape observed through a 3D laser scanning microscope (Keyence, VK-X1000). (b) The surface profile of microlens surface to measure radius of curvature. (c) The measured radius of curvature according to the lens diameter. The radius of curvature is linearly increased depending on the lens diameter in a fixed f-number.

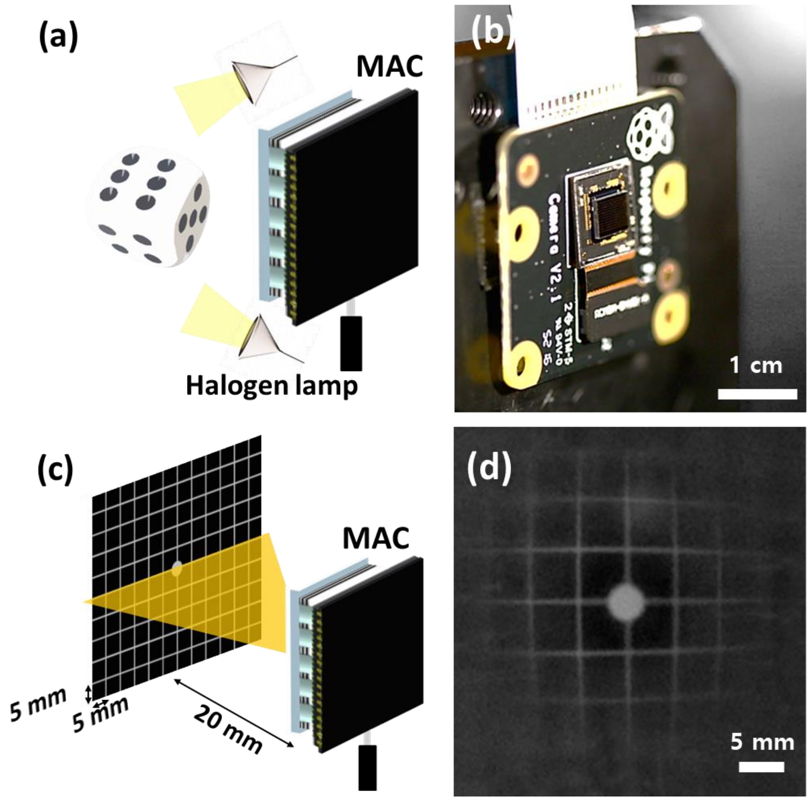


**Supplementary Fig. 3** Experimental setup and FOV measurement of MAC. (a) The experimental setup for assorted functional MAC imaging. The MAC with a printed circuit board was fixed to an optical mount. (b) A photograph of fully-assembled ultrathin camera. (c) A schematic illustration for measuring the camera FOV. (d) A captured grid object image through the single channel of MAC (The single-channel FOV: 73 degrees.

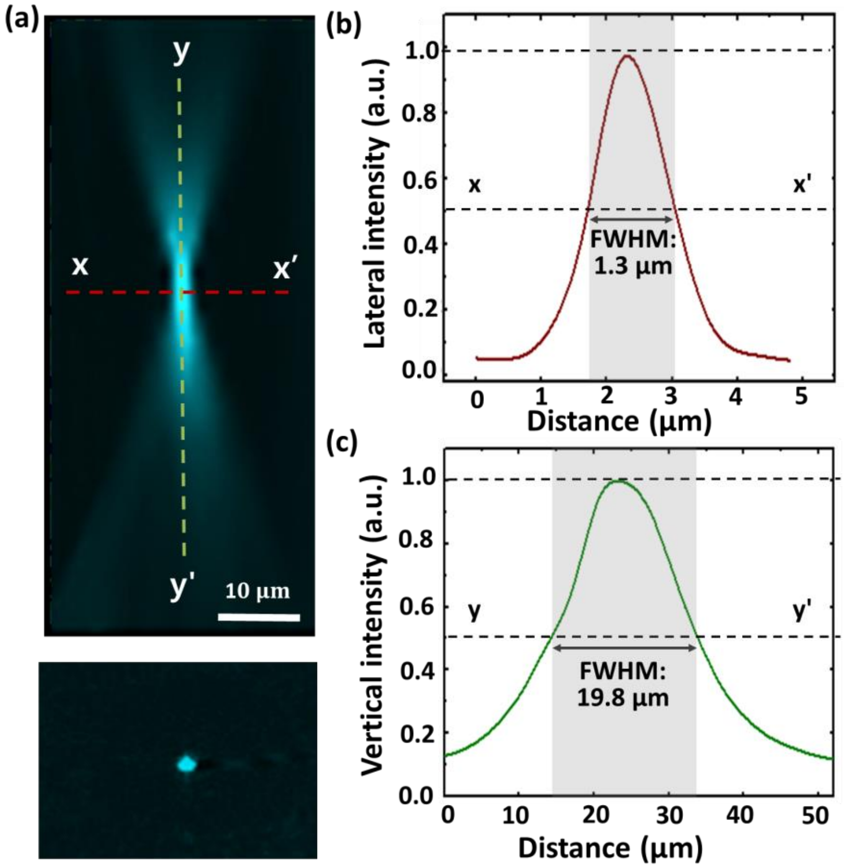


**Supplementary Fig. 4** Optical beam sectioning through a confocal laser scanning microscope. (a) Optical sections of 532 nm laser beam focusing passing through the iMLAs observed by a confocal laser scanning microscopy (CLSM). The full width at half maximum (FWHM) of (b) beam diameter and (c) the depth-of-focus measured from intensity profiles along line xx′ and yy′ on the sectioned image.

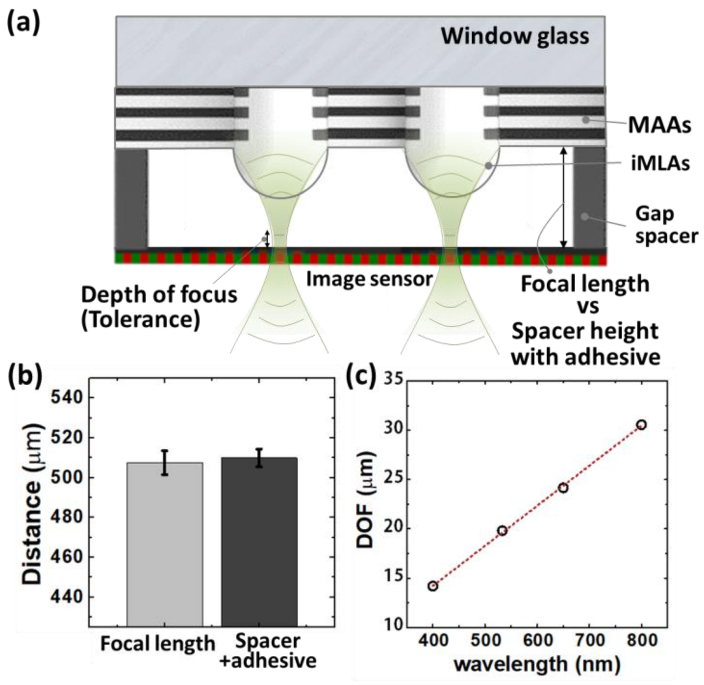


**Supplementary Fig. 5** Packaging tolerance of MAC. (a) A schematic illustration for explaining the packaging tolerance without the image blurring. The difference between the height of spacers with epoxy glue and the focal length should be less than the DOF of iMLAs. (b) Measured focal length (λ: 400~800 nm) and the height of spacers with epoxy glue. The error bars indicate the minimum and maximum range of repeated measurements. (c) Calculated Rayleigh range according to the wavelength in visible to near-infrared range. The depth of focus (DOF, Rayleigh range x 2) at 400 nm is 14.18 μm, which also allows the fabricated spacers tolerance of 12 μm.

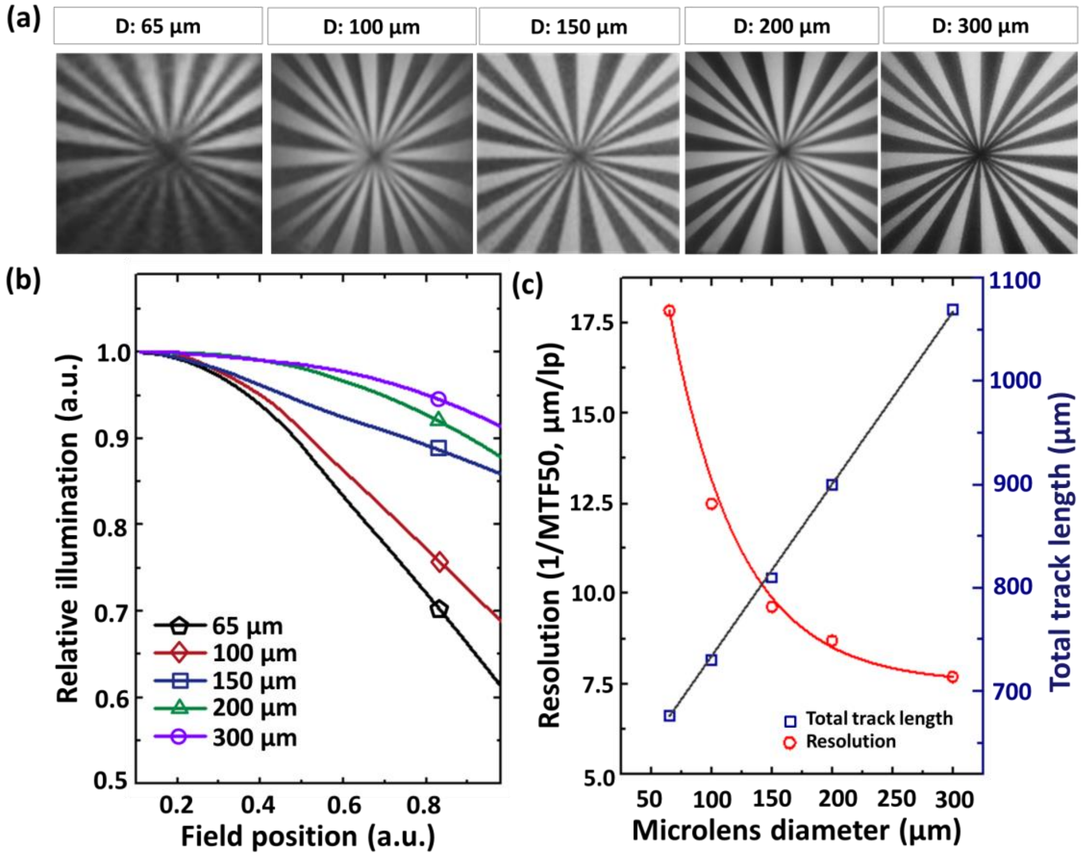


**Supplementary Fig. 6** Measured resolution of MAC. (a) Captured radial star images depending on the microlens diameter of iMLAs. (b) The corresponding relative illumination of radial star images measured from the center to the outside. (c) Image resolution (1/MTF50) and total track length depending on the lens diameter.

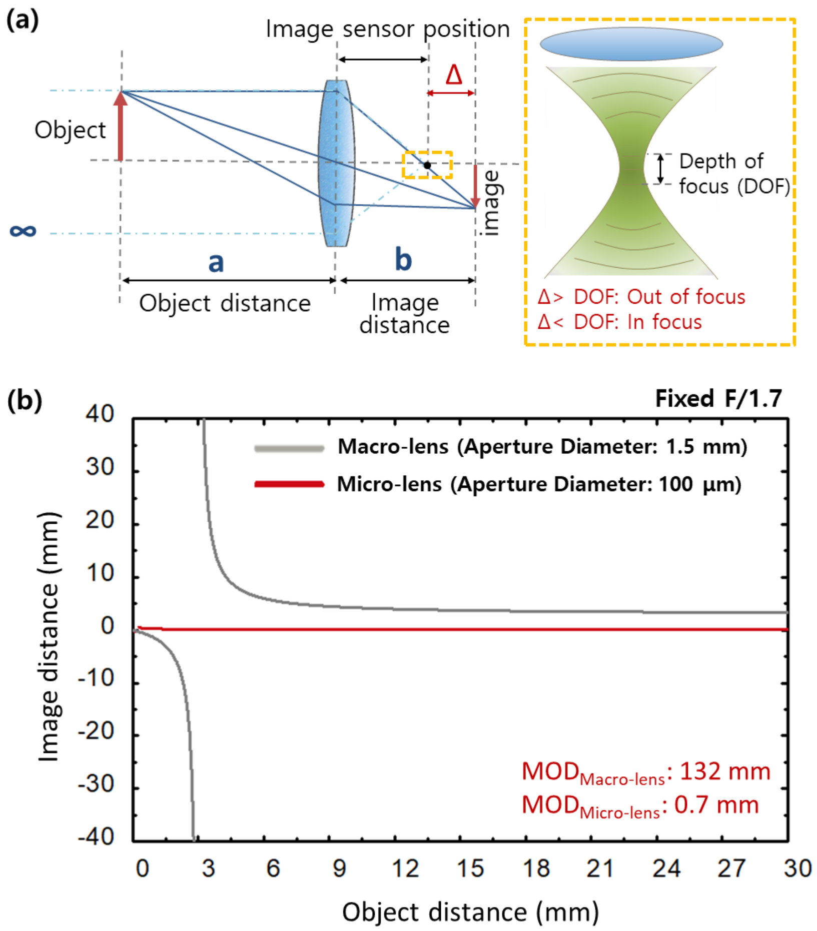


**Supplementary Fig. 7** Numerical analysis to estimate the minimum object distance (MOD). (a) Ray diagram and DOF schematics to explain the image formation. The difference (Δ) between image distance and image sensor position causes poorly focused images. The difference smaller than the DOF improves image focus. (b) The graph of lens equation for MOD measurement. The image distance in the macro-lens increases sharply at close object distance. However, the variation of image distance in the micro-lens is insignificant to reduce image focus. The calculated MOD result of the macro-lens is 132 mm, and that of the micro-lens is 700 μm.

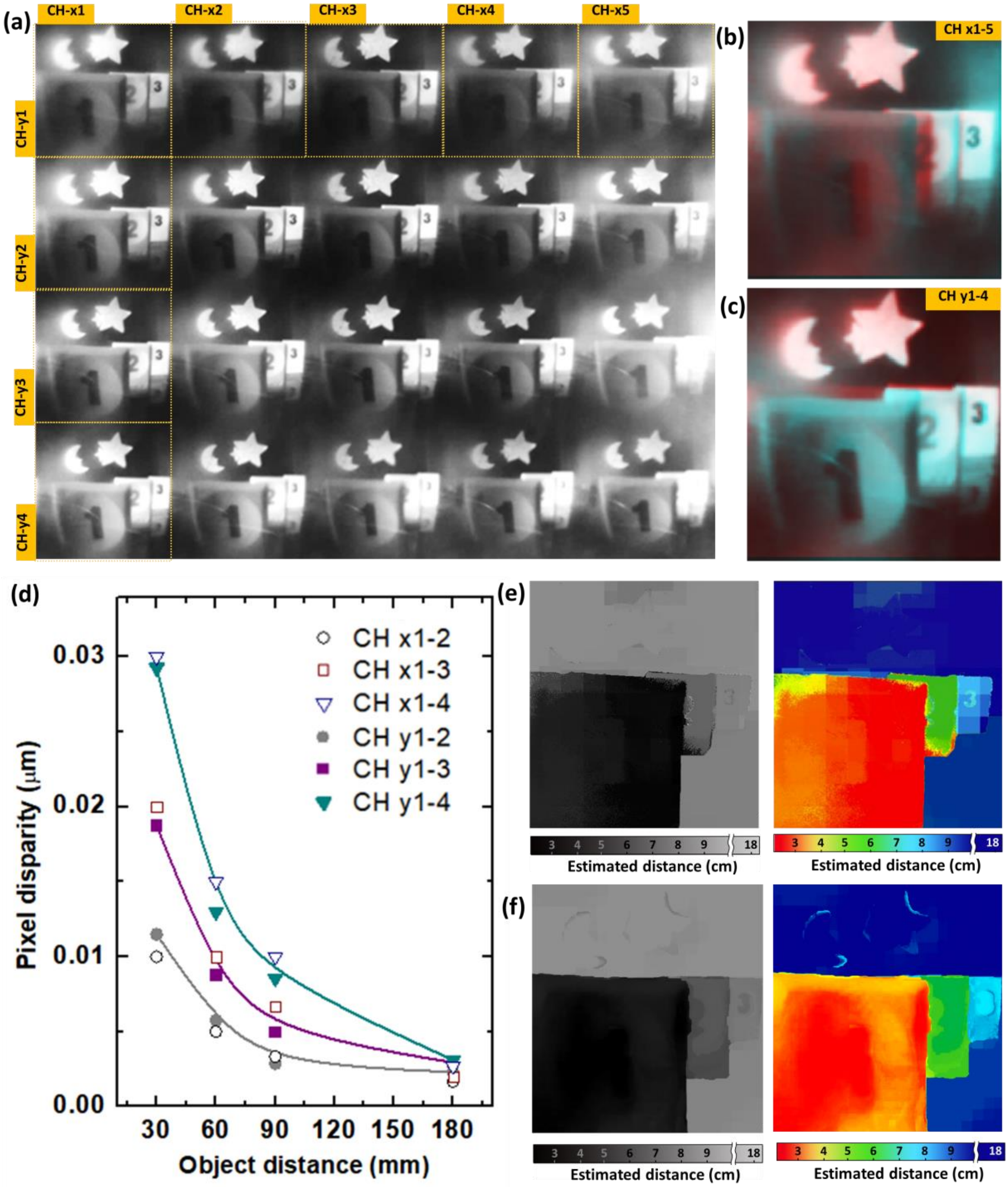


**Supplementary Fig. 8** 3D depth map through the MAC. (a) The array images of blocks with the number, a star, and a moon. The overlap differences of the number '2' clearly represent the visual disparities of each channel. Red-cyan anaglyph images integrated by (b) x-axis and (c) y-axis channel images, respectively. (d) Pixel disparity according to the object locations and the channel period on the x- and y-axis. (e, f) Reconstructed 3D depth images corresponding to images b and c.

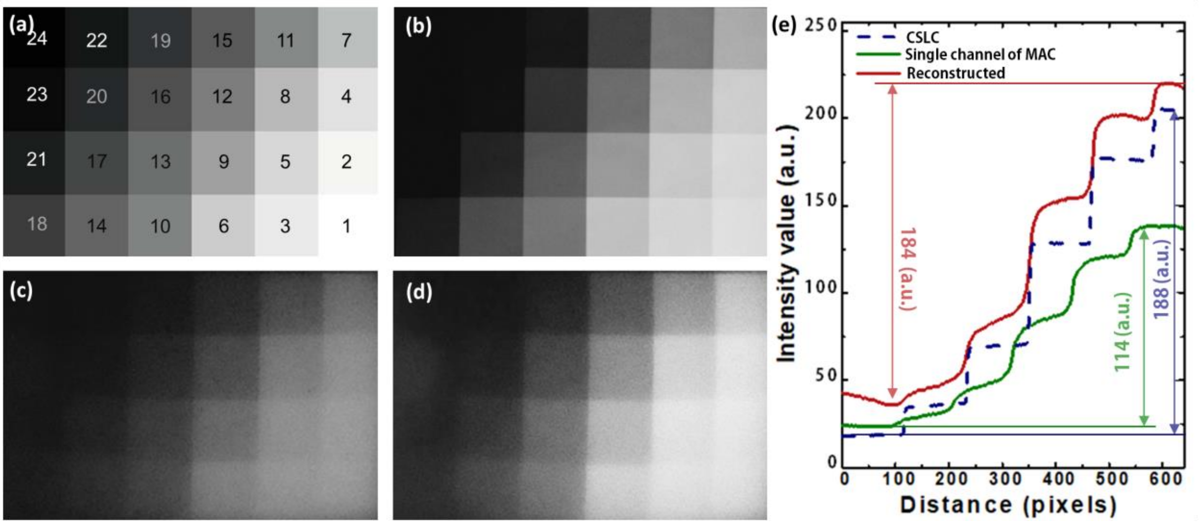


**Supplementary Fig. 9** Dynamic range measurement through ISO 21550 chart. (a) Reference image of ISO 21550 Dynamic range target. The target image captured by (b) the conventional single lens camera (CSLC, Histogram of Stdev.: 74.79) and (c) the single channel of MAC (Histogram of Stdev.: 46.85). (d) Reconstructed image from array images (Histogram of Stdev.: 75.13). (e) The corresponding normalized intensity surface profiles. The difference in intensity value of reconstructed image is increased by 1.61 times than that of single channel image through the HDR merge algorithm.

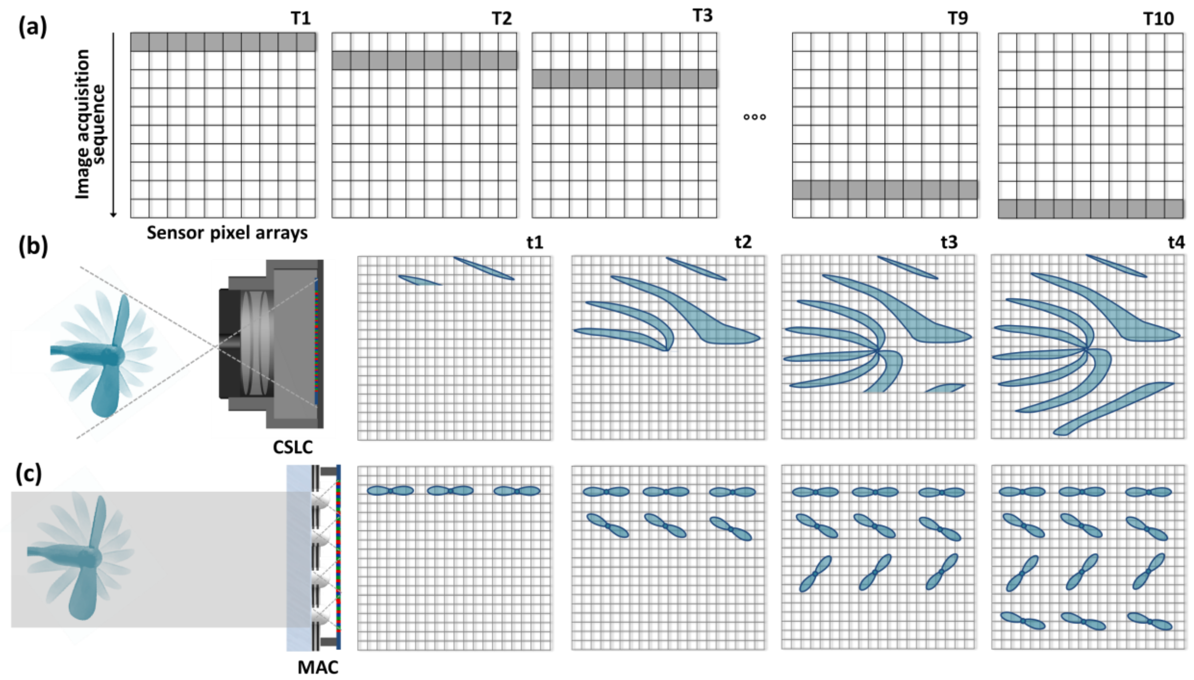


**Supplementary Fig. 10** Working principle of high-speed imaging. (a) Schematic illustrations for the image acquisition method of rolling shutter image sensor. A rolling shutter image sensor captures a single scene by scanning across pixels in vertical directions. (b) The imaging principle of acquiring fast-moving objects through the CSLC. The CSLC exhibits motion artifacts when the speed of object movement is faster than the image acquisition time. (c) The imaging principle of acquiring fast-moving objects through the MAC. The MAC captures multiple array images in the single sensor, which reduces the time to acquire images of each channel.

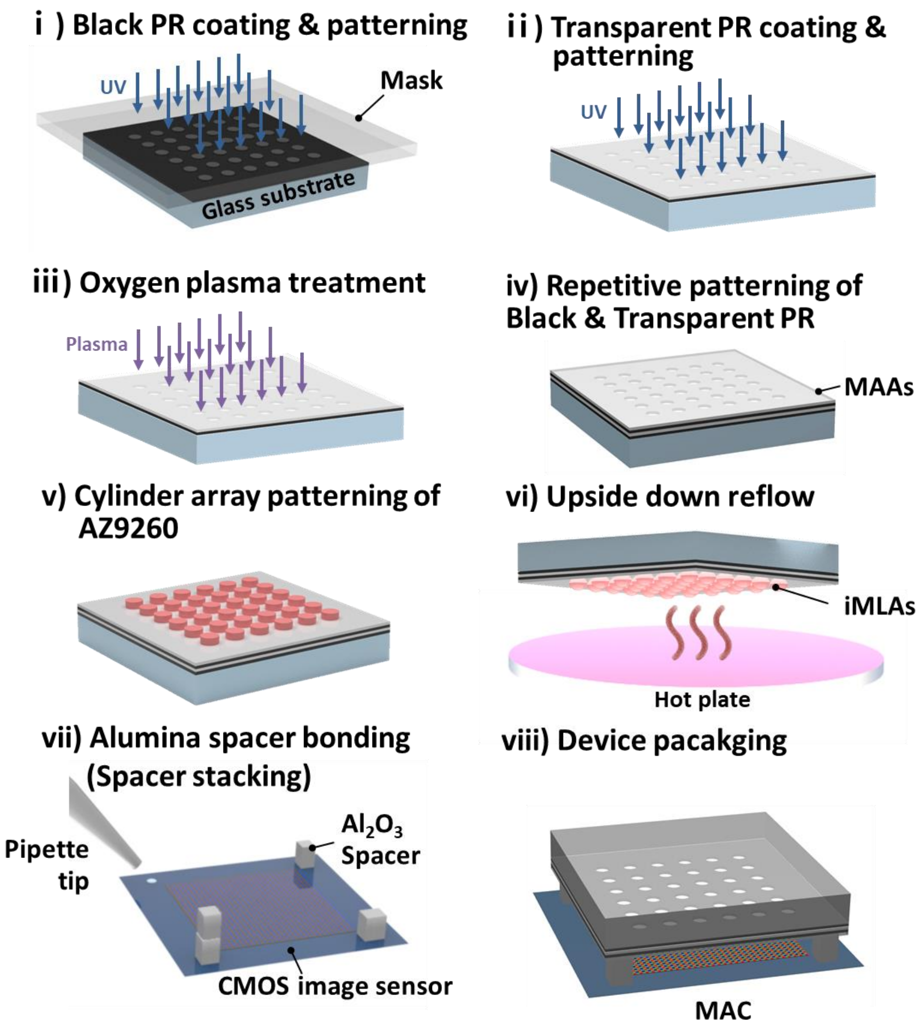


**Supplementary Fig. 11** Microfabrication steps for MAC. The ultrathin camera was fabricated through repetitive photolithography, upside-down reflow, and device packaging. The shape of microlens was formed by upside-down reflow to fabricate a lens without sagging on the microlens surface. 150 μm thick alumina spacers were vertically stacked to match the focal length with the image sensor position.
